# Primary healthcare competencies needed in the management of person-centred integrated care for chronic illness and multimorbidity: Results of a scoping review

**DOI:** 10.1186/s12875-023-02050-4

**Published:** 2023-04-12

**Authors:** Leslie Michielsen, Erik W.M.A. Bischoff, Tjard Schermer, Miranda Laurant

**Affiliations:** 1 School of Health Studies, HAN University of Applied Sciences, Nijmegen, the Netherlands; 2 Department of Primary and Community Care, Radboud Institute for Health Sciences, Radboud University Medical Centre, Nijmegen, The Netherlands; 3 Science Support Office, Gelre Hospitals, Apeldoorn, The Netherlands; 4 Radboud University Medical Centre, Radboud Institute for Health Sciences, IQ healthcare, Nijmegen, the Netherlands

**Keywords:** Primary care, Competencies, Chronic illness, Multimorbidity, Person-centred integrated care and interprofessional collaboration

## Abstract

**Background:**

Chronic disease management is important in primary care. Disease management programmes focus primarily on the respective diseases. The occurrence of multimorbidity and social problems is addressed to a limited extent. Person-centred integrated care (PC-IC) is an alternative approach, putting the patient at the centre of care. This asks for additional competencies for healthcare professionals involved in the execution of PC-IC. In this scoping review we researched which competencies are necessary for healthcare professionals working in collaborative teams where the focus lies within the concept of PC-IC. We also explored how these competencies can be acquired.

**Methods:**

Six literature databases and grey literature were searched for guidelines and peer-reviewed articles on chronic illness and multimorbidity in primary care. A data synthesis was carried out resulting in an overview of the competencies that healthcare professionals need to deliver PC-IC.

**Results:**

Four guidelines and 21 studies were included and four core competencies could be derived through the synthesis: 1. interprofessional communication, 2, interprofessional collaborative teamwork, 3. leadership and 4. patient-centred communication. Included papers mostly lack a clear description of the competencies in terms of knowledge, skills and attitudes which are necessary for a PC-IC approach and on how these competencies can be acquired.

**Conclusion:**

This review provides insight on competencies necessary to provide PC-IC within primary care. Research is needed in more depth on core concepts of these competencies which will then benefit educational programmes to ensure that healthcare professionals in primary care are better equipped to deliver PC-IC for patients with chronic illness and multimorbidity.

**Supplementary Information:**

The online version contains supplementary material available at 10.1186/s12875-023-02050-4.

## Background

Chronic diseases such as cardiovascular- and pulmonary disease and diabetes mellitus type 2 are the leading causes of death and disability worldwide. According to the World Health Organization these diseases kill 41 million people each year, equivalent to 71% of all deaths globally [1]. Approximately one in three adults suffer from more than one chronic disease [2, 3]. This is called multimorbidity, which is defined as the coexistence of two or more chronic conditions in the same individual [3].

The management of chronic diseases and multimorbidity is complex and the challenge is recognized worldwide. Patients with multimorbidity are at higher risk of safety issues for instance due to polypharmacy, more frequent and complex medication interactions and the involvement of different healthcare professionals resulting in competing priorities and lack of coordination of care [4]. Within the Health Education Framework (2017) it is stated: “A one-size-fits-all health care system simply cannot meet the increasing complexity of people’s needs and expectations” [5]. A broader perspective on the management of chronic disease seems necessary. A dominant focus on medical treatment is too limited, as the disease affects the daily living of patients [6]. Therefore it is argued that treatment programmes should include other domains of life as well, to meet the specific needs of individuals resulting in greater satisfaction with care and the physical and social well-being of patients [7].

This perspective has led to the development of personalised strategies to replace disease-management strategies, which can be referred to as Person-centred Integrated care (PC-IC) [5]. Person-centred care or Patient-centred care means that individuals’ values and preferences are elicited and that these preferences guide all aspects of their health care [8]. According to the HEE (Health Education England) Framework Patient- centred care means that people feel free to speak out about what is important to them and the healthcare professional listens to what matters to people [5].

There is no unifying definition or common conceptual understanding of integrated care due to the fact that there are different perspectives that construct the concept [9], However, there is consensus that integrated care is an approach to overcome care fragmentations, especially where this fragmentation and the disconnect between the different healthcare providers is leading to an adverse impact on people’s care experiences and care outcomes [9]. Integrated care is suitable for people with complex or long-term care needs.

In this review we use the term person-centred integrated care as an umbrella term comprising person-centred or patient-centred care and integrated care, as this refers to the holistic, individualized approach, empowering the patient to make effective care plans together with their healthcare providers, who collaborate interprofessionally, and patients as an equal partner. PC-IC is believed to improve outcomes and experience for persons with long-term and complex conditions [10].

Multimorbidity is predominantly dealt with in primary care [11]. The PC-IC approach of giving patients more choice and control in their lives is particularly suitable in this setting where general practitioners (GPs) often have a life-long relationship with patients [12]. Specific disease management programmes improve quality of care and patient outcomes in chronic disease [13]. However considering the complexity of care for patients with one or more chronic diseases their care needs often cannot be met by one single professional as different areas of expertise are necessary to optimize care for this large group of patients [6]. The primary care team consists of different professionals such as GPs, nurses, physical therapists, psychologists and dieticians who work side by side and rely on each other’s expertise and where necessary collaborate with professionals from other sectors, for instance hospitals and social welfare organizations. Involved healthcare professionals should be equipped to be a part of a collaborative, interprofessional team where the focus lies within the concept of PC-IC. It requires a specific skillset from team members. Being a member of such a collaborative team means working together and jointly setting achievable goals, which are based on the needs and preferences of the individual patient. Shifting from regular disease management towards PC-IC also means a shift in professional competencies due to the holistic approach that underlies it, which considers the different domains of the patient’s life. A competency is defined as an observable ability of a health professional, integrating multiple components such as knowledge, skills, values, and attitudes [14].

It is however still unclear which competencies these primary healthcare professionals should have or obtain in order to be able to deliver PC-IC in the primary care setting. In this scoping review our primary objective was to provide an overview of the current scientific knowledge on which competencies healthcare professionals who provide PC-IC to patients with one or more chronic disease should have. Our second aim was to get insight into how these competencies can be acquired.

## Methods

### Study design

We performed a scoping review guided by the methodological framework proposed by Arksey and O’Malley [15]; (I) identifying the research question, (II) identifying relevant studies, (III) selection of eligible studies, (IV) charting the data, and (V) collating, summarizing and reporting the results. A scoping review does not typically involve quality assessment of the methodology of empirical studies but are specifically designed to identify gaps in the evidence base [15]. We did not perform a critical appraisal based on study design as we aimed to include all available evidence.

#### I. identifying the research question

Our primary research question for the literature review was: Which interprofessional competencies do primary care professionals need to offer person-centred integrated care for patients with one or more chronic diseases? Our secondary research question was: How can these competencies be acquired?

#### II. Identifying relevant studies

We developed a comprehensive search strategy with the assistance of a librarian (TP) of the HAN University of Applied Sciences. The search included an extensive search string using Boolean operators and truncations to combine all relevant keywords and we checked the results of our search strategy against key publications. We chose a sensitive search strategy rather than a specific strategy, to ensure we would not miss relevant guidelines or peer-reviewed papers of our interest. Different definitions and concepts were included in the search string. For instance, multimorbidity and comorbidity were both added as they both refer to multiple chronic conditions (MCC). The difference we found is on how healthcare systems view patients with MCC. A hospital setting mostly looks at the one disease and then the comorbidities whereas the primary care setting or other generalist setting can easily change focus according to patient’s priorities [16]. There is also a variation in the terminology used to describe team collaboration; terms include ‘multidisciplinary’, ‘interdisciplinary’, ‘interprofessional’ and ‘multiprofessional’. The term interprofessional applies when two or more professions learn or practice together to improve health outcomes in patients whereas multiprofessional applies when professions practice together but not necessarily on shared goals [17]. The search was conducted from onset of the respective literature databases till September 2020. In January 2023 we updated our search, using the same search strategy, to see if there were any new articles or guidelines that could be added to this scoping review. First, we searched for chronic disease guidelines and chronic disease management programmes that involved the primary care setting. The search for the guidelines took place in in the Trip medical database (https://www.tripdatabase.com) with the following terms including their linguistic variations; (a) primary care, (b) integrated care, (c) chronic illness, (d) multimorbidity, (e) shared decision making and (f) competencies (Appendix 1). For this search no filters were applied. Next, using the same keywords, we searched for peer-reviewed articles in the following scientific literature databases: Cinahl, Embase, PubMed, Medline, and Web of Science (Appendix 1). Grey literature was hand-searched through websites of relevant national and international journals, scanning reference lists and through Google and Google Scholar by the main researcher (LM). We included all literature without date restrictions. We only searched for articles written in English or Dutch as these languages were covered by the authors. Search records were downloaded, combined and de-duplicated using EndNote bibliographic software (Clarivate Analytics, Philadelphia, PA, U.S.A.). Afterwards, we exported our search records to Rayyan QCRI which facilitates process of blind screening [18]. All titles, abstracts and full texts were reviewed against inclusion and exclusion criteria, see Table 1.

**Table 1:** Inclusion and exclusion criteria

	Illness/condition	Setting	outcome	Study design/guidelines
**Inclusion criteria**	Pulmonary disease; Cardiovascular disease; Obesity; Palliative or end-of-life (with a life expectancy > 6 months) All publicationsreporting on chronic diseases, multimorbidity or comorbidity, without reporting a specific diagnosis	Primary healthcare	Competencies, attitude, skills and knowledge for healthcare professionals delivering person-centred and integrated care Strategies for healthcare professionals attaining competencies	All types of empirical studies. Practice guidelines and disease management programmes for chronic disease(s)
**Exclusion criteria**	Illnesses where treatment mostly takes place in hospital or specialized care facility like cancer or mental illness. Terminal care (life expectancy < 6 months) because there is no curative treatment or long-term goal setting. Publications that only focus on diagnostic and/or pharmacology for chronic illnesses Paediatric care or publications focused on chronic care for children.	Hospital or specialized care facility	Other outcomes (e.g. clinical outcomes, patient satisfaction, process of care, resource utilization etc.) or skills for patients	Conference abstracts Conference posters Study protocols

#### III. Study selection

The titles and abstracts of both the guidelines and peer-reviewed articles were screened blind by pairs of two out of four researchers (LM, AT, EB, ML, NvD) of which the main researcher (LM) screened all identified guidelines and peer-reviewed articles.

First the titles and abstracts were screened for relevance. Publications considered relevant only by one of the two reviewers were discussed until consensus was reached. Secondly the full text publications were read, and data were extracted by one author and checked by a second. We included published, peer-reviewed and grey literature. All types of study designs describing competencies could be included.

#### IV. Charting the data

Two reviewers (LM, ML) jointly developed a data charting form in Excel to describe relevant information. One reviewer extracted data from the included empirical studies and guidelines. The form included information on [1] study design, [2] country, [3] aim or objective, [4] participants and [5] the described competencies.

The main researcher (LM) filled in the data forms, which were subsequently checked by one of the other researchers (ML or EB). The authors frequently met to discuss the charting of the data. At the first stage of analysis, we collected descriptions of any statement potentially related to the competencies for the execution of PC-IC excluding disease specific competencies. This resulted in four overarching themes. At the second stage of the analysis one reviewer identified the underlying core concepts, i.e., skills, knowledge and attitudes that emerged under the four main themes These were then summarised under the interdependent themes. The extracted details were cross-checked by a second researcher (ML). She read all the notes and coding of the first researcher (LM) and cross-checked this against the papers and guidelines. If any discrepancy was discovered, this was discussed until consensus was reached.

#### V. Collating, summarizing and reporting the results

In this final step a narrative report was produced to summarize the extracted data. The Prisma checklist for scoping reviews was used to make sure we covered all essential items [19].

## Results

**Fig. 1:**
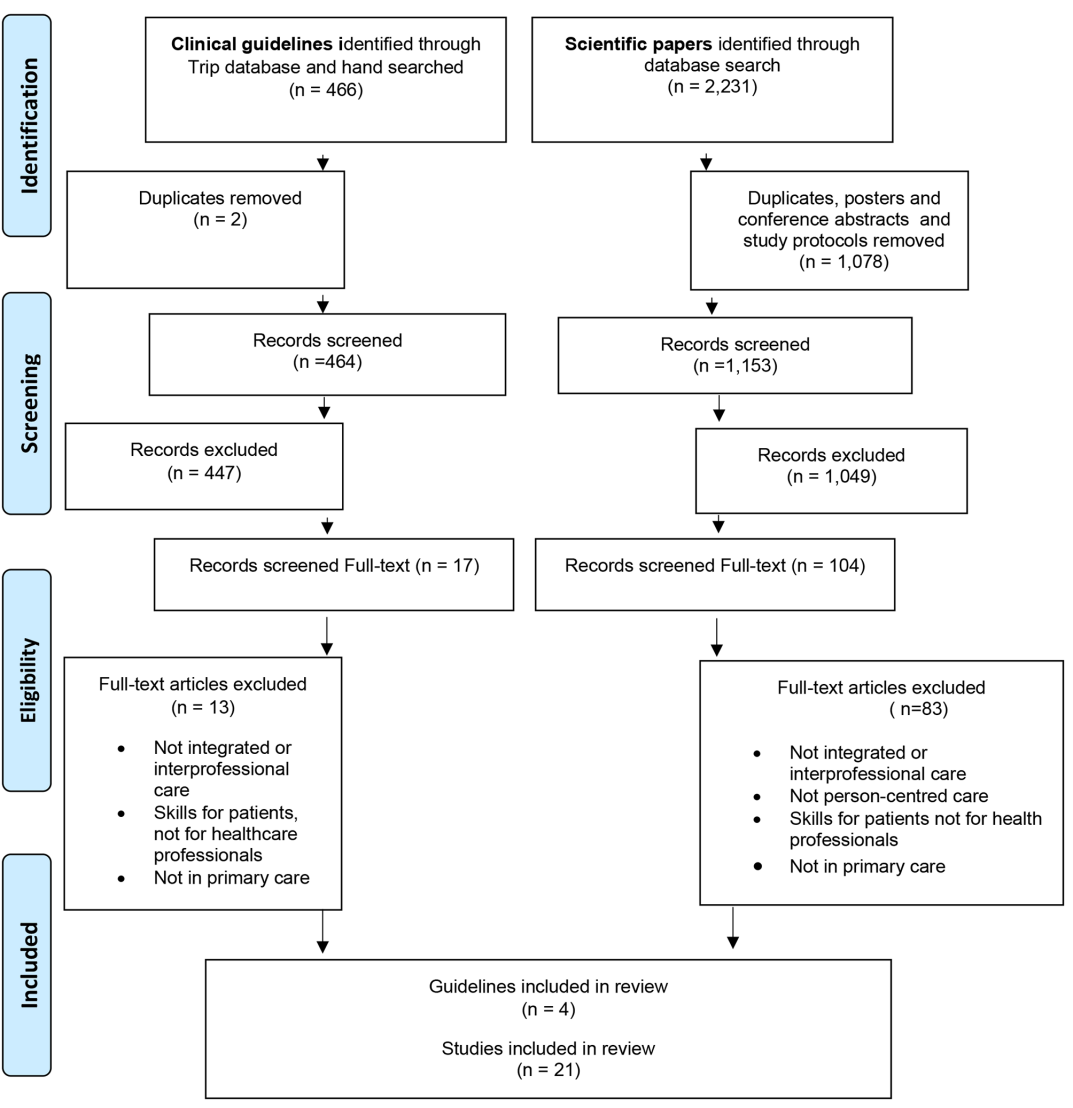
Flow chart describing the process of the review of clinical guidelines and scientific peer-reviewed articles

The initial searches identified 327 guidelines and 1,810 articles. In January 2023 the search was updated which resulted in 139 guidelines and 421 new articles to be screened. After removing duplicates, posters and conference abstracts a total of 464 guidelines and 1,153 articles were screened for inclusion (Fig. 1). Disagreements were solved in discussion between the two researchers, and it was not necessary to include a third researcher as referee. The screening resulted in 17 guidelines and 104 articles which were selected for full text review. After reading the full text publications, a total of 4 guidelines and 21 articles met our inclusion criteria and therefor were included in the data synthesis.

### Study characteristics

Table 2 reports the study characteristics of the included studies and guidelines. The four guidelines included were from United States (n = 2), Australia (n = 1) and Switzerland (n = 1). Publication dates ranged between 2014 and 2021. The guidelines covered different patient populations, one was on COPD (chronic obstructive pulmonary disease) [20], one on elderly people [21], one on Palliative and End of Life care in stroke patients [22], and one on primary prevention of chronic disease in the general practice setting [23]. The 21 included peer-reviewed papers used quantitative, qualitative and mixed research methods. The designs varied from one randomized controlled trial [24], four literature reviews [25–28], two expert opinions [29, 30], and two studies were mixed methods studies [31, 32]. The remaining twelve studies were qualitative studies [33–44]. The included studies were performed in the United States (n = 9), the Netherlands (n = 5), Australia (n = 2) and one study in each of the following countries: Belgium, Canada, Ireland, New Zealand and the United Kingdom. Publication dates ranged between 2006 and 2020.

**Table 2:** Characteristics of included studies and guidelines

1st author/Organisation and year of publication	Country	Aim/objective	Participants	Theme	Code/Subtheme
Primary study: Qualitative study					
Abramowitz et al. 2010	USA	The gap in training, a curriculum for a Primary Care Internal Medicine residency programme that links a practical form of motivational interviewing training to the self-management support component of the chronic care model was developed and piloted.	Primary care internal residents	Person-centred communication	Motivational interviewing
Aerts et al. 2019	Belgium	The views of general practitioners, practice nurses and patients on interprofessional collaboration in general practice and to understand to what extent the nurse-doctor relationship meets their needs and expectations.	Nurses	Person-centred communication Interprofessional communication Collaborative teamwork	Open communication Motivational interviewing Open communication Equality Clarity of roles Teamwork Trust and respect
Al Hamayel et al. 2018	USA	Older patients’ perspectives on the quality of serious illness care in primary care	Primary healthcare clinicians	Person-centred communication Collaborative teamwork	Open communication Listening Sharing information
Byrne et al. 2019	Australia	How nurse navigators manage client care.	Nurse navigator	Interprofessional communication Leadership	Open communication Team leadership
DeJesus et al. 2012	USA	What qualities do patients and providers look for in a care manager.	Care manager	Person-centred communication Collaborative teamwork	Open communication Motivational interviewing Nonverbal communication Listening Motivation
van Dijk-de Vries et al. 2012	The Netherlands	The views of Dutch stakeholders on achieving a biopsychosocial approach to the care of patients with chronic diseases.	Primary care providers	Person-centred communication Collaborative teamwork	Nonverbal communication Listening Clarity of roles Teamwork
van Dongen et al. 2016	The Netherlands	Influential factors regarding interprofessional collaboration related to care plan development in primary care.	Primary care providers	Person-centred communication Collaborative teamwork Leadership	Person-centred assessment Open communication Shared language Knowing each other Trust and respect Motivation Sharing information Team leadership Team meetings Care advocate
Dudley et al. 2018	USA	Understanding the facilitators and barriers to optimal, coordinated interdisciplinary provision of community-based palliative care.	Primary care providers and palliative care	Person-centred communication Interprofessional communication Collaborative teamwork	Open communication Two way communication Clarity of roles
Fouche et al. 2014	New Zeeland	The perspectives of New Zealand healthcare practitioners from seven professional groups involved in chronic care (general practice medicine, nursing, occupational therapy, pharmacy, physiotherapy, social work, and speech language therapy) on the core competencies required of those working in this area.	Primary healthcare professionals involved in chronic care	Person-centred communication Interprofessional communication Collaborative teamwork	Clarity of communication Two way communication Sharing information Knowing each other Clarity of roles Teamwork
Mercer et al. 2016	UK/Ireland	Development and optimising a primary care-based complex intervention (CARE Plus) to enhance the quality of life of patients with multimorbidity in the deprived areas.	Primary care providers	Person-centred communication	Person-centred assessment Listening
Lawn et al. 2009	Australia	The skills required by primary health care professionals to provide effective chronic condition prevention and self-management support, according to the perceptions of a sample of Australian consumers and carers.	Primary care providers	Person-centred communication Interprofessional communication Collaborative teamwork	Motivational Interviewing Open communication Listening Two way communication Knowing each other Sharing information
Van de Pol et al. 2017	The Netherlands	Identifying core competencies for shared decision making with frail older persons, and the second is to determining key elements of a teaching framework, based on the authors’ recently developed model for shared decision making with older patients who are frail.	Healthcare professionals involved with the care for frail older patients	Person-centred communication Collaborative teamwork	Person-centred assessment Sharing information
Primary study: RCT					
Helitzer et al. 2010	USA	Efficacy and effectiveness of training to improve primary care providers’ Person-centred communication skills and proficiency in discussing their patients’ health risks.	Primary care providers	Person-centred communication	Person-centred assessment
Primary study: Mixed methods					
Hillebregt et al. 2016	The Netherlands	Barriers and facilitators influencing self-management among COPD patients.	Primary care providers	Person-centred communication	Open communication Equality
Lenzen et al. 2018	The Netherlands	How a shared decision-making approach was implemented and experienced by practice nurses and patients.	Nurses	Person-centred communication Interprofessional communication	Open communication Listening Open communication
Secondary study: Review					
Anstiss 2009 (Literature review)	USA	A description of Motivational Interviewing, where it comes from, evidence of its effectiveness and how its potential might be better realised.	Primary care providers	Person-centred communication	Motivational Interviewing Listening
Dale et al. 2016 (Narrative review)	United Kingdom	Integrated care models that incorporate behavioural health care are part of the solution in primary care.	Behavioural health consultants	Person-centred communication Interprofessional communication Collaborative teamwork Leadership	Motivational Interviewing Sharing information Clarity of roles Team leadership
Fowler et al. 2020 (Literature review)	USA	The value of IP^1^ team-based care, continuing professional development, and the impact of the team on practice performance and health outcomes.	Primary care providers	Collaborative teamwork Leadership	Clarity of roles Shared language Teamwork Team leadership
Golden et al. 2019 Review	USA	Models that provide key elements of integrated biopsychosocial care for persons with serious illness effectively and cost efficiently.	Community based organisations	Interprofessional collaboration Collaborative teamwork	Two way communication Clarity of roles Trust and respect Sharing information
Lein et al. 2006 Review	USA	Effective and efficient Person-centred interviewing strategies to enhance the management of complex primary care patient encounters.	Nurse practitioners	Person-centred communication	Motivational Interviewing Person-centred assessment Nonverbal communication
Rocker et al. 2015 Review	Canada	The concept of primary palliative care as a more holistic, person-centred approach.	Primary palliative care	Person-centred communication	Open communication Person-centred assessment
Guidelines					
World Health Organisation 2019	Switzerland	Guideline for integrated health and social care for the implementation of the ICOPE approach (integrated care for older people.	Community based informal and formal care for older people	Person-centred communication Collaborative teamwork	Person-centred assessment Teamwork
The Royal Australian College of General Practitioners (RACGP) 2018	Australia	Guideline for the implementation of prevention in the general practice setting.	General practitioners and practice nurses	Person-centred communication Interprofessional communication Collaborative teamwork Leadership	Person-centred assessment Motivational Interviewing Open communication Knowing each other Team leadership
Heart Association/American Stroke Association 2020	USA	Primary palliative care competencies and skills to be considered, learned, and practiced by providers and healthcare services when caring for patients and families with stroke.	Healthcare providers involved in primary palliative care	Person-centred communication Interprofessional communication Collaborative teamwork Leadership	Person-centred assessment Listening Effective communication Sharing information Teamwork Team leadership
Department of Defense 2014	USA	Guideline on the management of Chronic Obstructive Pulmonary Disease, intended to assist primary care providers.	Primary care providers	Person-centred communication Collaborative teamwork	Open communication Teamwork

^1^ Interprofessional.

The specific healthcare professionals involved in the execution of PC-IC varied. Seven studies involved PC-IC from the perspective of one profession: nurses [32, 35, 36], nurse practitioners [30], general practitioners [34], behavioral health consultants [27], or primary care internal medicine residents [33]. Three studies involved a mix of healthcare professionals including general practitioners, nurses, occupational therapists, pharmacists, physiotherapists, social workers and speech language therapists [24, 41, 42]. In the remaining eleven studies the authors did not specify the profession (25– [26, 28]– [29, 31, 37–40, 43]–44). The scope of the studies involved different patient populations: patients with multimorbidity [25, 30, 39, 42], frail elderly or elderly with serious illness [29, 34, 44], multimorbidity or aging population [27], palliative care (28 40), prevention of chronic illness [24, 43], or COPD [31]. In nine studies the chronic illness was not specified (26–27, 32, 33, 35–38, 41). All studies involved a form of person-centred care described as ‘a whole person approach’, ‘shared decision making’ or ‘improving self-management’.

### Identified competencies

All competencies concerning PC-IC as described in the included documents were extracted. The data synthesis identified four main themes: 1* patient-centred communication 2* interprofessional communication; 3* collaborative teamwork and 4* Leadership. In Appendix 2 we report the code tree with examples from included studies.

### Person-centred competencies

#### Person-centred communication

All guidelines [20–23] and 18 articles [20–24, 26–28, 30–35, 37–44] describe professional’s communication with patients to be an important competency within PC-IC. Open communication is central to person-centred care (20, 28, 31– [32, 34–37, 39]–40, 43). Communication with patients should also be based on equality [31, 35]. Professionals with good communication skills conduct person-centred assessments to identify what matters most to the patient [21–24, 28, 30, 39, 42, 44]. In patient-centred communication professionals support their messages by evidence-based information tailored to the patient’s needs [20]. Professionals should also be skilled in relational communication techniques for communication with caregiver(s), family members or a delegated decision-maker [22, 28]. Good listening skills are strongly highlighted within the PC-IC approach (22, 26, 32, 34, 37– [38, 42]–43). Professionals should recognize nonverbal signals and strive for clarity of communication 30, 37–38). It is important that professionals take the level of understanding due to, for instance, language barriers, physical impairments and possible cultural differences into consideration (21–22, 38, 41, 44). They also respond to patient’s emotions and needs and follow-up by providing tailored responses to these needs [22, 24, 34]. Furthermore, professionals should be able to apply motivational interviewing techniques, as research has shown that this improves the quality of professional – patient interaction and shared decision making (23, 26–27, 30, 33, 35, 37, 43).

### Interprofessional competencies

#### Interprofessional communication

Two guidelines [22, 23] and 8 articles described communication to be an important competency when offering PC-IC (27, 29, 32, 35– [36, 40]–41, 43). Communication requires a two way and open dialoge between professionals, in team meetings as well as in bilateral conversations (27, 32, 35–36, 39–41, 43)Decision-making, problem-solving and goal setting are important issues to be discussed with each other (22–23, 34, 39). Also, this should be an interdisciplinary team effort (21, 38, 41–42, 44). It is essential that the collaborating healthcare professionals are able to discover shared patient goals during team meetings (21, 23, 27, 35, 39– [40]–41). Each healthcare professional should have the ability to communicate with colleagues and other disciplines in a bidirectional manner [39, 43]. This means that each party is aware of the other’s professional backgrounds, strengths and boundaries and points in which professionals can reinforce each other. Team consensus is reached by dialoguing and discussing issues with all team members on an equal level [35]. In the communication own professional perspectives and expertise are highly valued and contribute to the quality of PC-IC plans [35]. Good communication skills are not only necessary within the primary care team, but it is equally important that these healthcare professionals show good communication skills towards external organizations such as other healthcare services or community agencies [20, 23, 41]. The heart association American stroke association [22] describes the importance of effective communication between professionals, but no further explanation what competences are needed for effective communication.

### Collaborative teamwork

All guidelines [20–23] and 12 articles (25, 27, 29, 34– [35, 37–41, 43]–44) described interprofessional teamwork or team collaboration skills. Healthcare professionals should have the ability and motivation to work collaboratively with others and share pertinent information [22, 27, 29, 34, 37, 39, 41, 44] and also important to share knowledge of each other’s involvement when sharing the same goals for their patients [30, 34, 40, 43]. Person-centred care is a team effort and is achieved through teamwork [20–22, 25, 35, 38, 41] Another critical competency is the intrinsic motivation of professionals to collaborate with others [37, 39]. This is essential as interprofessional collaboration is often considered to be time consuming, while time is scarce. Interpersonal factors may also cause barriers to collaboration and therefore it is important to define a shared language and discuss the diversity of personal perspectives [25, 39]. Healthcare professionals should know who else is on the team and there should be a clear understanding of the professional’s own roles as well as a clear understanding of the other profession’s roles and competencies [23, 27, 29, 39–41, 43]. It could be helpful if the professionals within the collaborative team invest in getting to know each other. Research has shown that professionals knowing each other well are better able to take advantage of each other’s discipline-specific competencies [39]. Knowing each other also contributes to an atmosphere of mutual trust and respect which creates an open and safe environment in which the professionals involved dare to think and act broader than their own discipline [23, 35, 39].

### Leadership

Two guideline (22–23) and four articles [25, 27, 36, 39] mark good leadership as an important competency for sustainable and effective collaboration in interprofessional teams. Team leadership characteristics include modelling and advocating of interprofessional teamwork, providing resources and infrastructure, and promoting shared team leadership, goals and decision making (22–23, 25, 27, 36, 39). Leadership skills are also required for bringing the interprofessional team together and to support professionals to adopt the shift in values and attitudes towards collaborative working [23, 25, 27, 39]. Leadership skills are also necessary for attaining efficient and successful team meetings (i.e., planning, agenda setting, structuring, chairing) [39]. Although all team members should have leadership skills, within the collaborative team one team member should take the role as leader or coordinator and monitor the team’s shared goals and objectives [23, 25, 39]. Professionals with strong leadership competencies show to be patient care advocates; they ensure that the team discusses the patient’s goals and needs and that patients are put in the centre of care [36, 39].

### Acquiring the competencies necessary to offer person-centred integrated care for patients with one or more chronic diseases

Three guidelines [21–23] and 17 articles (24–29, 32–33, 35, 37–44) mentioned the need for ongoing education or training for professionals, either for communication, interprofessional collaboration or for the execution of the PC-IC approach. This requires new knowledge and skills, but a change in attitude is also necessary. Most articles considered education to be a major facilitating factor to ensure that (future) professionals are equipped to provide care for patients with chronic illness and multimorbidity. Professional education to develop knowledge and skills should be incorporated in undergraduate programmes as well as in postgraduate programmes and be part of on-the-job training (35, 41–42). In interprofessional education two or more professions learn with, about, and from each other to enable effective collaboration and improve health outcomes in patients [21–23, 25]. Learning together with other healthcare professionals will also improve the understanding of each other’s roles [29, 41]. Two papers specified the training needs. Van der Pol et al. [44] and Helitzer et al. [24] reported that professionals need specific training on communication. In particular professionals need more skills in asking open ended questions. Rocker et al. [28] emphasized that during medical training, by effective mentorship and observation, medical students should obtain in depth skills on how to discover patient’s needs.

## Discussion

This scoping review identified and described interprofessional competencies as well as patient-centred competencies which are needed when professionals aim to provide PC-IC in primary care. The overall findings contained limited information about specific qualifications and competencies. The descriptions of the competencies are mostly described as general competencies for instance; ‘communication skills’ and are rarely defined in detail. The HEE framework describes in more detail which competencies are shown when a professional delivers person-centred care. The aim of the framework is to set out core, transferable behaviours, knowledge and skills [5]. With regard to communicative competencies, we also found some details, similar to the HEE framework, such as asking open-ended questions but just asking open ended questions does not make that a healthcare professional delivers person-centred care. Asking open-ended questions to explore and understand the patient, his or her personal situation and what matters to him or her does make it more person-centred [5]. We did not find details on how the competencies can be trained. Nonetheless, we were able to derive important competencies from the findings. Communication, collaborative teamwork and leadership seem to be essential competencies that healthcare professionals in primary care should either have or make sure to acquire when delivering PC-IC.

The communication competencies that would be expected from healthcare professionals apply to interprofessional communication as well as to patient-centred communication, and both should be based on equality and respect for the interlocutor(s). This is also confirmed by a recent literature review on competencies to promote collaboration between primary and secondary care physicians [45]. This particular review also showed, similar to our findings, that team members should be open minded and willing to look beyond one’s own position [45]. We found that healthcare professionals should know who else is on the team and there should be a clear understanding of the other profession’s roles and competencies. Knowing each other also contributes to an atmosphere of mutual trust and respect. Perceived hierarchy is the main conceptual barrier hindering collaboration between professionals. A new approach leads to a shift from subordination to complementarity in order to meet patients’ needs [46] and to strengthen interprofessional collaboration. Patient-centred care requires physicians and other healthcare professionals to have communication skills to elicit patients’ true wishes and to recognize and respond to both their needs and emotional concerns [47]. As described in the HEE framework the workforce listens to what matters to the patients and giving them the opportunity to speak out freely [5]. Our findings show that asking open ended questions, listening, recognizing nonverbal signs and the ability to adjust to the level of understanding of the patient are the most important communication skills needed to accomplish this.

We also found that leadership skills are needed to facilitate interprofessional collaboration in more than one way. Leadership skills are needed by professionals within the primary care setting, but also in relation to collaboration with professionals from external organizations. Jansen et al. [45] described three levels on which leadership can be demonstrated; 1* in relation with other persons, 2* to facilitate collaboration, and 3* showing leadership at a system level to create an environment in which primary and secondary care collaboration is promoted and facilitated.

In the included articles the factor ‘time’ is important to facilitate interprofessional collaboration and the execution of PC-IC. Time is important during consultation in order to build a relationship with the patient and meet their needs (23, 30, 39–40, 42–44). The lack of time and the large number of patients to see daily are important barriers when dealing with patients with multimorbidity. Other research also shows that seeing more than 3 or 4 patients per hour may lead to suboptimal content of consultations, lower patient satisfaction, increased patient turnover, or inappropriate prescribing [48]. This points to the direction that, besides competencies, also a different way of practice organization (extra consultation time) is necessary for successful execution of PC-IC (39–40, 43, 44). Besides time for patient consultations, the current payment systems may hinder collaboration between healthcare professionals as interprofessional meetings are often not reimbursed [39].

In preparing health care professionals to take on this task, establishing standards for training in PC-IC is important. The HEE framework describes core, transferable behaviours, knowledge and skills for becoming a person-centred healthcare professional. The framework focus on communicative competences and interventions that can be implemented. It also describes learning outcomes which can be used to educate healthcare professionals. However the scope of this framework is not specific to a certain practice and additional content therefor may be required for some roles and context [5]. Our findings can be seen as the additional content, specifically in the context of the primary care practice. The prevalence of chronic illness is growing worldwide, and management is increasingly undertaken by interprofessional teams, yet education is still generally provided monodisciplinary [34]. Educational training of both undergraduate as well as graduated healthcare professionals is needed to better prepare healthcare professionals to meet the needs of ageing patients with multiple chronic conditions in a way that is person-centred, effective and sustainable [49]. Patients’ personal goals can be used as a guide in interprgessionl collaboration as it might have the porential to integrat different care plans with each other [50]. However, there is still a need for professionals to acquire competencies to discuss patients’ personal goals through training [50]. Interprofessional education has an important role to play in professionals developing the competencies required to collaborate successfully [47]. Future research on education should guide professionals in acquiring different qualifications and competencies.

### Strengths and Limitations

To our knowledge this is the first review to provide an overview of competencies that healthcare professionals should possess to deliver PC-IC in primary care. Another strength of our review is that we used various and broad search terms, allowing inclusion of all types of literature, both scientific and grey. The aim of this study is to provide a comprehensive list of competencies. We deliberately chose to include all types of study designs and guidelines without limitations in order to capture relevant guidelines as well as scientific articles.

This study was also subject to some limitations. We excluded studies in languages other than English and Dutch. Although we might have missed some studies, most studies are likely to be published in English. While performing this review, we noted rather heterogenous terminology describing the concept of the PC-IC approach as well as for interprofessional collaboration. Therefore, to optimize our search strategy we thoroughly explored different definitions and concepts before finalizing the search strategy. Nonetheless we may have missed relevant studies that report PC-IC related competencies due to the use of different terminology.

According to guidelines for scoping review we did not undertake a methodology quality assessment of the included articles, although critical appraisal of methodology and ranking the evidence by level of evidence is commonly used in systematic reviews and meta-analysis of the literature. We deliberately chose to include all types of study designs and guidelines without limitations in order to capture all required competencies. We gave equal weight to all included guidelines and articles, regardless of the robustness of the underlying methodology. We consider this justified given the purpose of the scoping study, i.e., providing a narrative account of competencies for executing PC-IC and how these can be acquired.

## Conclusion

We identified interprofessional as well as patient care-related competencies to be relevant for the execution of person-centred integrated primary healthcare. Nonetheless, guidelines and articles mostly lack a detailed description of the competencies in terms of skills, knowledge and attitudes. Insight in these core concepts are necessary to properly educate healthcare professionals in primary care to deliver PC-IC. Further research in which the core concepts of the required competencies are clearly described is still necessary to properly prepare primary healthcare professionals to offer high value care to patients with chronic diseases and multimorbidity. Educational programmes, both undergraduate and postgraduate, should take these competencies into account. A shift towards interprofessional education is necessary to acquire these competencies.

## Electronic supplementary material

Below is the link to the electronic supplementary material.

Supplementary Material 1

Supplementary Material 2

Supplementary Material 3

## Data Availability

All data generated or analysed during this study are included in this published article.

## Abbreviations

COPD: Chronic Obstructive Pulmonary Disease.
ICOPE: Integrated care for older people.
PC-IC: Person-centred integrated care.
