# Supplementary material for: Primary healthcare competencies needed in the management of person-centred integrated care for chronic illness and multimorbidity: Results of a scoping review
Source: BMC Prim Care. 2023 Apr 12;24:98. doi: 10.1186/s12875-023-02050-4 (PMC10091550; doi:10.1186/s12875-023-02050-4)
Supplement: Supplementary file 1 — Supplementary Material 1 [file 12875_2023_2050_MOESM1_ESM.docx]

1. Noncommunicable diseases: World Health Organisation; 2021 Available from: <https://www.who.int/news-room/fact-sheets/detail/noncommunicable-diseases>.

2. Hajat C, Stein E. The global burden of multiple chronic conditions: A narrative review. Prev Med Rep. 2018 Oct 19;12:284-293. doi: 10.1016/j.pmedr.2018.10.008. PMID: 30406006; PMCID: PMC6214883.

3. Academy of medical sciences. Multimorbidity: a priority for global health research. The Academy of Medical Sciences, 2018. Available from: [82222577 (acmedsci.ac.uk)](https://acmedsci.ac.uk/file-download/82222577)

4. K Barnett, S Mercer, M Norbury, G Watt, S Wyke, B Guthrie

The epidemiology of multimorbidity in a large cross-sectional dataset: implications for health care, research and medical education

Lancet, 380 (2012), pp. 37-43

5. Person-Centred Approaches: Empowering people in their lives and communities to enable an upgrade in prevention, wellbeing, health, care and support. A core skills education and training framework. Health Education England (HEE); 2017.

6. Grimsmo A, Løhre A, Røsstad T, Gjerde I, Heiberg I, Steinsbekk A. Disease-specific clinical pathways - are they feasible in primary care? A mixed-methods study. Scand J Prim Health Care. 2018 Jun;36(2):152-160. doi: 10.1080/02813432.2018.1459167. Epub 2018 Apr 12. PMID: 29644927; PMCID: PMC6066276.

7. What is patient centred care and why is it important? United Kingdom: Health Innovation Network; 2021 Available from: <http://healthinnovationnetwork.com/system/ckeditor_assets/attachments/41/what_is_person-centred_care_and_why_is_it_important.pdf>

8. American Geriatrics Society Expert Panel on Person-Centered Care. Person-Centered Care: A Definition and Essential Elements. J Am Geriatr Soc. 2016 Jan;64(1):15-8. doi: 10.1111/jgs.13866. Epub 2015 Dec 2. PMID: 26626262.

9. World health Organisation. Integrated care models: an overview. Health Services Delivery Programme Division of Health Systems and Public Health. 2016 Available from: [Integrated care models: an overview (who.int)](https://www.euro.who.int/__data/assets/pdf_file/0005/322475/Integrated-care-models-overview.pdf?source=post_page---------------------------)

10. Berntsen G, Høyem A, Lettrem I, Ruland C, Rumpsfeld M, Gammon D. A person-centered integrated care quality framework, based on a qualitative study of patients' evaluation of care in light of chronic care ideals. BMC Health Serv Res. 2018 Jun 20;18(1):479. doi: 10.1186/s12913-018-3246-z.

11. Muth C, van den Akker M, Blom JW, Mallen CD, Rochon J, Schellevis FG, et al. The Ariadne principles: how to handle multimorbidity in primary care consultations. BMC Med. 2014 Dec 8;12:223. doi: 10.1186/s12916-014-0223-1. PMID: 25484244; PMCID: PMC4259090.

12. Cocksedge S, Greenfield R, Nugent GK, Chew-Graham C. Holding relationships in primary care: a qualitative exploration of doctors' and patients' perceptions. Br J Gen Pract. 2011 Aug;61(589):e484-91. doi: 10.3399/bjgp11X588457. PMID: 21801542; PMCID: PMC3145532.

13. Dennis SM, Zwar N, Griffiths R, Roland M, Hasan I, Powell Davies G, Harris M. Chronic disease management in primary care: from evidence to policy. Med J Aust. 2008 Apr 21;188(S8):S53-6. doi: 10.5694/j.1326-5377.2008.tb01745.x. PMID: 18429737.

14. Frank JR, Snell LS, Cate OT, Holmboe ES, Carraccio C, Swing SR, Harris P, Glasgow NJ, Campbell C, Dath D, Harden RM, Iobst W, Long DM, Mungroo R, Richardson DL, Sherbino J, Silver I, Taber S, Talbot M, Harris KA. Competency-based medical education: theory to practice. Med Teach. 2010;32(8):638-45. doi: 10.3109/0142159X.2010.501190. PMID: 20662574.

15. Arksey H, O'Malley L, Scoping Studies: Towards a Methodological Framework. International Journal of Social Research Methodology. 2005 (Theory & Practice, 8(1), 19–32).

16. Harrison C, Fortin M, van den Akker M, Mair F, Calderon-Larranaga A, Boland F, Wallace E, Jani B, Smith S. Comorbidity versus multimorbidity: Why it matters. J Multimorb Comorb. 2021 Mar 2;11:2633556521993993. doi: 10.1177/2633556521993993. PMID: 33718251; PMCID: PMC7930649.

17. Gilbert JH, Yan J, Hoffman SJ. A WHO report: framework for action on interprofessional education and collaborative practice. J Allied Health. 2010 Fall;39 Suppl 1:196-7. PMID: 21174039

18. Ouzzani M, Hammady H, Fedorowicz Z, & Elmagarmid A, Rayyan—a web and mobile app for systematic reviews. Systematic Reviews. 2016;5(1):210.

19. Peters MDJ, Marnie C, Tricco AC, Pollock D, Munn Z, Alexander L, et al. Updated methodological guidance for the conduct of scoping reviews. JBI Evid Synth. 2020 Oct;18(10):2119-2126. doi: 10.11124/JBIES-20-00167. PMID: 33038124.

20. VA/DoD Clinical Practice Guideline for the management of chronic obsructive pulmonary disease (2014). Washington (DC): Department of Veterans Affairs, Department of Defense.

21. World Health Organization. (‎2019)‎. Integrated care for older people (‎ICOPE)‎ implementation framework: guidance for systems and services. World Health Organization. https://apps.who.int/iris/handle/10665/325669. License: CC BY-NC-SA 3.0 IGO

22. Holloway RG, Arnold RM, Creutzfeldt CJ, et al. Palliative and end-of-life care in stroke: a statement for healthcare professionals from the American Heart Association/American Stroke Association. Stroke. 2014;45(6):1887-1916. doi:10.1161/STR.0000000000000015

23. Putting prevention into practice: a guide for the implementation of prevention in the general practice setting. Putting prevention into practice. The Royal Australian College of General Practitioners. 2018.

24. Helitzer DL, Lanoue M, Wilson B, de Hernandez BU, Warner T, Roter D. A randomized controlled trial of communication training with primary care providers to improve patient-centeredness and health risk communication. Patient Educ Couns. 2011 Jan;82(1):21-9. doi: 10.1016/j.pec.2010.01.021. Epub 2010 Mar 12. PMID: 20219315; PMCID: PMC3539754.

25. Fowler T, Garr D, Mager NDP, Stanley J. Enhancing primary care and preventive services through Interprofessional practice and education. Isr J Health Policy Res. 2020 Mar 23;9(1):12. doi: 10.1186/s13584-020-00371-8. PMID: 32204734; PMCID: PMC7092466.

26. Anstiss T. Motivational interviewing in primary care. J Clin Psychol Med Settings. 2009 Mar;16(1):87-93. doi: 10.1007/s10880-009-9155-x. Epub 2009 Mar 1. PMID: 19253016.

27. Dale H, Lee A. Behavioural health consultants in integrated primary care teams: a model for future care. BMC Fam Pract. 2016 Jul 29;17:97. doi: 10.1186/s12875-016-0485-0. PMID: 27473414; PMCID: PMC4966805.

28. Rocker GM, Simpson AC, Horton R. Palliative Care in Advanced Lung Disease: The Challenge of Integrating Palliation Into Everyday Care. Chest. 2015 Sep;148(3):801-809. doi: 10.1378/chest.14-2593. PMID: 25742140.

29. Golden RL, Emery-Tiburcio EE, Post S, Ewald B, Newman M. Connecting Social, Clinical, and Home Care Services for Persons with Serious Illness in the Community. J Am Geriatr Soc. 2019 May;67(S2):S412-S418. doi: 10.1111/jgs.15900. PMID: 31074858.

30. Lein C, Wills CE. Using patient-centered interviewing skills to manage complex patient encounters in primary care. J Am Acad Nurse Pract. 2007 May;19(5):215-20. doi: 10.1111/j.1745-7599.2007.00217.x. PMID: 17489953.

31. Hillebregt CF, Vlonk AJ, Bruijnzeels MA, van Schayck OC, Chavannes NH. Barriers and facilitators influencing self-management among COPD patients: a mixed methods exploration in primary and affiliated specialist care. Int J Chron Obstruct Pulmon Dis. 2016 Dec 23;12:123-133. doi: 10.2147/COPD.S103998. PMID: 28096666; PMCID: PMC5214516.

32. Lenzen SA, Daniëls R, van Bokhoven MA, van der Weijden T, Beurskens A. What makes it so difficult for nurses to coach patients in shared decision making? A process evaluation. Int J Nurs Stud. 2018 Apr;80:1-11. doi: 10.1016/j.ijnurstu.2017.12.005. Epub 2017 Dec 16. PMID: 29331655.

33. Abramowitz SA, Flattery D, Franses K, Berry L. Linking a motivational interviewing curriculum to the chronic care model. J Gen Intern Med. 2010 Sep;25 Suppl 4(Suppl 4):S620-6. doi: 10.1007/s11606-010-1426-6. PMID: 20737238; PMCID: PMC2940440.

34. Abu Al Hamayel N, Isenberg SR, Hannum SM, Sixon J, Smith KC, Dy SM. Older Patients' Perspectives on Quality of Serious Illness Care in Primary Care. Am J Hosp Palliat Care. 2018 Oct;35(10):1330-1336. doi: 10.1177/1049909118771675. Epub 2018 Apr 22. PMID: 29682975.

35. Aerts N, Van Bogaert P, Bastiaens H, Peremans L. Integration of nurses in general practice: A thematic synthesis of the perspectives of general practitioners, practice nurses and patients living with chronic illness. J Clin Nurs. 2020 Jan;29(1-2):251-264. doi: 10.1111/jocn.15092. Epub 2019 Nov 28. PMID: 31713277.

36. Byrne AL, Hegney D, Harvey C, Baldwin A, Willis E, Heard D, et al. Exploring the nurse navigator role: A thematic analysis. J Nurs Manag. 2020 May;28(4):814-821. doi: 10.1111/jonm.12997. Epub 2020 Apr 16. PMID: 32155679.

37. Dejesus RS, Vickers KS, Howell LA, Stroebel RJ. Qualities of care managers in chronic disease management: patients and providers' expectations. Prim Care Diabetes. 2012 Oct;6(3):235-9. doi: 10.1016/j.pcd.2012.03.003. Epub 2012 Apr 23. PMID: 22534170.

38. van Dijk-de Vries A, Moser A, Mertens VC, van der Linden J, van der Weijden T, van Eijk JT. The ideal of biopsychosocial chronic care: how to make it real? A qualitative study among Dutch stakeholders. BMC Fam Pract. 2012 Mar 12;13:14. doi: 10.1186/1471-2296-13-14. PMID: 22405260; PMCID: PMC3355054.

39. van Dongen JJ, Lenzen SA, van Bokhoven MA, Daniëls R, van der Weijden T, Beurskens A. Interprofessional collaboration regarding patients' care plans in primary care: a focus group study into influential factors. BMC Fam Pract. 2016 May 28;17:58. doi: 10.1186/s12875-016-0456-5. PMID: 27233362; PMCID: PMC4884411.

40. Dudley N, Ritchie CS, Rehm RS, Chapman SA, Wallhagen MI. Facilitators and Barriers to Interdisciplinary Communication between Providers in Primary Care and Palliative Care. J Palliat Med. 2019 Mar;22(3):243-249. doi: 10.1089/jpm.2018.0231. Epub 2018 Nov 1. PMID: 30383468.

41. Fouche C, Kenealy T, Mace J, Shaw J. Practitioner perspectives from seven health professional groups on core competencies in the context of chronic care. J Interprof Care. 2014 Nov;28(6):534-40. doi: 10.3109/13561820.2014.915514. Epub 2014 May 14. PMID: 24828623.

42. Mercer SW, O'Brien R, Fitzpatrick B, Higgins M, Guthrie B, Watt G, et al. The development and optimisation of a primary care-based whole system complex intervention (CARE Plus) for patients with multimorbidity living in areas of high socioeconomic deprivation. Chronic Illn. 2016 Sep;12(3):165-81. doi: 10.1177/1742395316644304. Epub 2016 Apr 10. PMID: 27068113; PMCID: PMC4995497.

43. Lawn S, Battersby M, Lindner H, Mathews R, Morris S, Wells L, et al. What skills do primary health care professionals need to provide effective self-management support? Seeking consumer perspectives. Australian Journal of Primary Health. 2009(15):37–44. DOI:10.1071/PY08053

44. van de Pol MHJ, Fluit CRMG, Lagro J, Slaats Y, Olde Rikkert MGM, Lagro-Janssen ALM. Shared decision making with frail older patients: Proposed teaching framework and practice recommendations. Gerontol Geriatr Educ. 2017 Oct-Dec;38(4):482-495. doi: 10.1080/02701960.2016.1276014. Epub 2016 Dec 27. PMID: 28027017.

45. Janssen M, Sagasser MH, Fluit CRMG, Assendelft WJJ, de Graaf J, Scherpbier ND. Competencies to promote collaboration between primary and secondary care doctors: an integrative review. BMC Fam Pract. 2020 Sep 2;21(1):179. doi: 10.1186/s12875-020-01234-6. PMID: 32878620; PMCID: PMC7469099.

46. Supper I, Catala O, Lustman M, Chemla C, Bourgueil Y, Letrilliart L. Interprofessional collaboration in primary health care: a review of facilitators and barriers perceived by involved actors. J Public Health (Oxf). 2015;37(4):716-27.

47. Levinson W. Patient-centred communication: a sophisticated procedure. BMJ Qual Saf. 2011 Oct;20(10):823-5. doi: 10.1136/bmjqs-2011-000323. Epub 2011 Aug 19. PMID: 21856961.

48. Dugdale DC, Epstein R, Pantilat SZ. Time and the patient-physician relationship. J Gen Intern Med. 1999;14 Suppl 1(Suppl 1):S34-S40. doi:10.1046/j.1525-1497.1999.00263.x

49. Miller R, Scherpbier N, van Amsterdam L, Guedes V, Pype P. Inter-professional education and primary care: EFPC position paper. Prim Health Care Res Dev. 2019 Oct 4;20:e138. doi: 10.1017/S1463423619000653. PMID: 31581968; PMCID: PMC6784359.
