# Supplementary material for: Primary healthcare competencies needed in the management of person-centred integrated care for chronic illness and multimorbidity: Results of a scoping review
Source: BMC Prim Care. 2023 Apr 12;24:98. doi: 10.1186/s12875-023-02050-4 (PMC10091550; doi:10.1186/s12875-023-02050-4)
Supplement: Supplementary file 2 — Supplementary Material 2 [file 12875_2023_2050_MOESM2_ESM.docx]

**Appendix 1**

**Embase 24-01-2023**

1 exp primary health care/ OR general practice/ OR (((General OR Primary OR famil*) ADJ1 (care OR healthcare OR medicine OR practice*)) OR (community ADJ1 based)). ti,ab,kw.

2 ((comprehensive OR coordinated OR Integrated OR seamless) ADJ1 (care OR healthcare)).ti,ab,kw.

3 exp multidisciplinary team/ OR ((Inter OR multi OR interagency*) ADJ1 (collaborat* OR team* OR cooperation*) OR Multidisciplinar* OR Interdisciplinar* OR ((Inter OR multi) ADJ1 (disciplinar* OR colaborat*)) OR (Collaborative ADJ1 practice*) OR IPC OR (Chronic ADJ1 (care OR healthcare) ADJ1 model) OR Network*).ti,ab,kw.

4 shared decision making/ OR (SDM OR (Shared ADJ1 decision ADJ1 making) OR (patient ADJ1 (centered OR centred) ADJ1 care) OR (patient ADJ1 (centeredness OR engagement))).ti,ab,kw.

5 2 OR 3 OR 4

6 comorbidity/ OR exp multiple chronic conditions/ OR (Multimorbidit* OR Comorbidit* OR (multiple ADJ1 chronic ADJ1 condition*) OR (Chronic ADJ1 (illness OR disease*)) OR "Multi-morbidity" OR (multiple ADJ1 chronic ADJ1 condition*)).ti,ab,kw.

7 clinical competence/ OR aptitude/ OR (aptitude* OR capabilit* OR competenc* OR expertise OR proficien* OR skill*).ti,ab,kw.

8 1 AND 5 AND 6 AND 7

9 limit 8 to conference abstract status

10 8 NOT 9

**Medline 24-01-2023**

S1 MH "Primary Health Care" OR MH "Family Practice" OR TI (((General OR Primary OR famil*) N1 (care OR healthcare OR medicine OR practice*)) OR (community N1 based)) OR AB (((General OR Primary OR famil*) N1 (care OR healthcare OR medicine OR practice*)) OR (community N1 based)) OR SU (((General OR Primary OR famil*) N1 (care OR healthcare OR medicine OR practice*)) OR (community N1 based))

S2 TI ((comprehensive OR coordinated OR Integrated OR seamless) N1 (care OR healthcare)) OR AB ((comprehensive OR coordinated OR Integrated OR seamless) N1 (care OR healthcare)) OR SU ((comprehensive OR coordinated OR Integrated OR seamless) N1 (care OR healthcare))

S3 MH "Patient Care Team" OR TI ((Inter OR multi OR interagenc*) N1 (collaborat* OR team* OR cooperation*) OR Multidisciplinar* OR Interdisciplinar* OR ((Inter OR multi) N1 (disciplinar* OR colaborat*)) OR (Collaborative N1 practice*) OR IPC OR (Chronic N1 (care OR healthcare) N1 model) OR Network*) OR AB ((Inter OR multi OR interagenc*) N1 (collaborat* OR team* OR cooperation*) OR Multidisciplinar* OR Interdisciplinar* OR ((Inter OR multi) N1 (disciplinar* OR colaborat*)) OR (Collaborative N1 practice*) OR IPC OR (Chronic N1 (care OR healthcare) N1 model) OR Network*) OR SU ((Inter OR multi OR interagenc*) N1 (collaborat* OR team* OR cooperation*) OR Multidisciplinar* OR Interdisciplinar* OR ((Inter OR multi) N1 (disciplinar* OR colaborat*)) OR (Collaborative N1 practice*) OR IPC OR (Chronic N1 (care OR healthcare) N1 model) OR Network*)

S4 MH "Decision Making, Shared" OR TI (SDM OR (Shared N1 decision N1 making) OR (patient N1 (centered OR centred) N1 care) OR (patient N1 (centeredness OR engagement))) OR AB (SDM OR (Shared N1 decision N1 making) OR (patient N1 (centered OR centred) N1 care) OR (patient N1 (centeredness OR engagement))) OR SU (SDM OR (Shared N1 decision N1 making) OR (patient N1 (centered OR centred) N1 care) OR (patient N1 (centeredness OR engagement)))

S5 S2 OR S3 OR S4

S6 MH "Comorbidity" OR TI (Multimorbidit* OR Comorbidit* OR (multiple N1 chronic N1 condition*) OR (Chronic N1 (illness OR disease*)) OR “Multi-morbidity” OR (multiple N1 chronic N1 condition*)) OR AB (Multimorbidit* OR Comorbidit* OR (multiple N1 chronic N1 condition*) OR (Chronic N1 (illness OR disease*)) OR “Multi-morbidity” OR (multiple N1 chronic N1 condition*)) OR SU (Multimorbidit* OR Comorbidit* OR (multiple N1 chronic N1 condition*) OR (Chronic N1 (illness OR disease*)) OR “Multi-morbidity” OR (multiple N1 chronic N1 condition*))

S7 MH "Clinical Competence+" OR MH "Aptitude" OR TI (aptitude* OR capabilit* OR competenc* OR expertise OR proficien* OR skill*) OR AB (aptitude* OR capabilit* OR competenc* OR expertise OR proficien* OR skill*) OR SU (aptitude* OR capabilit* OR competenc* OR expertise OR proficien* OR skill*)

S8 S1 AND S5 AND S6 AND S7

**PubMed 24-01-2023**

#1 "Primary Health Care"[mesh] OR "Family Practice"[mesh] OR Primary care[tiab] OR primary health care[tiab] OR General medicine[tiab] OR General healthcare[tiab] OR General practice[tiab] OR Family practice[tiab] OR family care[tiab] OR family practice[Mesh] OR community based[tiab] OR primary practice*[tiab]

#2 Integrated care[tiab] OR integrated healthcare[tiab] OR coordinated care[tiab] OR comprehensive care[tiab] OR seamless care[tiab]

#3 "Patient Care Team"[Mesh:NoExp] OR patient care team*[ti] OR Interprofessional collaboration[tiab] OR (intersectoral[tiab] AND teamwork[tiab]) OR interagency cooperation[tiab] OR multidisciplinary[tiab] OR interdisciplinary[tiab] OR Collaborative practice*[tiab] OR IPC[tiab] OR Intersectoral Collaboration[MeSH Terms] OR Chronic care model[tiab] OR chronic care[tiab] OR network[tiab]

#4 "Decision Making, Shared"[Mesh] OR SDM[tiab] OR Shared decision making[tiab] OR patient centered care[tiab] OR patient centeredness[tiab] OR patient engagement[tiab]

#5 #2 OR #3 OR #4

#6 "Comorbidity"[Mesh] OR multimorbidit*[tiab] OR Comorbidit*[tiab] OR multiple chronic condition*[tiab] OR Chronic illness[tiab] OR chronic disease[tiab] OR Multi-morbidity[tiab] OR multiple chronic condition*[tiab]

#7 Clinical Competence[MeSH Terms] OR "Aptitude"[Mesh] OR aptitude*[tiab] OR capabilit*[tiab] OR competenc*[tiab] OR expertise[tiab] OR proficienc*[tiab] OR skill*[tiab]

#8 #1 AND #5 AND #6 AND #7

**Cochrane 24-01-2023**

#1 (((General OR Primary OR famil*) NEAR/1 (care OR healthcare OR medicine OR practice*)) OR (community NEAR/1 based)):ti,ab,kw

#2 ((comprehensive OR coordinated OR Integrated OR seamless) NEAR/1 (care OR healthcare)):ti,ab,kw

#3 ((Inter OR multi OR interagenc*) NEAR/1 (collaborat* OR team* OR cooperation*) OR Multidisciplinar* OR Interdisciplinar* OR ((Inter OR multi) NEAR/1 (disciplinar* OR colaborat*)) OR (Collaborative NEAR/1 practice*) OR IPC OR (Chronic NEAR/1 (care OR healthcare) NEAR/1 model) OR Network*):ti,ab,kw

#4 (SDM OR (Shared NEAR/1 decision NEAR/1 making) OR (patient NEAR/1 (centered OR centred) NEAR/1 care) OR (patient NEAR/1 (centeredness OR engagement))):ti,ab,kw

#5 #2 OR #3 OR #4

#6 (Multimorbidit* OR Comorbidit* OR (multiple NEAR/1 chronic NEAR/1 condition*) OR (Chronic NEAR/1 (illness OR disease*)) OR "Multi-morbidity" OR (multiple NEAR/1 chronic NEAR/1 condition*)):ti,ab,kw

#7 (aptitude* OR capabilit* OR competenc* OR expertise OR proficien* OR skill*):ti,ab,kw

#8 #1 AND #5 AND #6 AND #7

**Cinahl 24-01-2023**

S1 MH "Primary Health Care" OR MH "Family Practice" OR TI (((General OR Primary OR famil*) N1 (care OR healthcare OR medicine OR practice*)) OR (community N1 based)) OR AB (((General OR Primary OR famil*) N1 (care OR healthcare OR medicine OR practice*)) OR (community N1 based)) OR SU (((General OR Primary OR famil*) N1 (care OR healthcare OR medicine OR practice*)) OR (community N1 based))

S2 TI ((comprehensive OR coordinated OR Integrated OR seamless) N1 (care OR healthcare)) OR AB ((comprehensive OR coordinated OR Integrated OR seamless) N1 (care OR healthcare)) OR SU ((comprehensive OR coordinated OR Integrated OR seamless) N1 (care OR healthcare))

S3 MH "Multidisciplinary Care Team" OR TI ((Inter OR multi OR interagenc*) N1 (collaborat* OR team* OR cooperation*) OR Multidisciplinar* OR Interdisciplinar* OR ((Inter OR multi) N1 (disciplinar* OR colaborat*)) OR (Collaborative N1 practice*) OR IPC OR (Chronic N1 (care OR healthcare) N1 model) OR Network*) OR AB ((Inter OR multi OR interagenc*) N1 (collaborat* OR team* OR cooperation*) OR Multidisciplinar* OR Interdisciplinar* OR ((Inter OR multi) N1 (disciplinar* OR colaborat*)) OR (Collaborative N1 practice*) OR IPC OR (Chronic N1 (care OR healthcare) N1 model) OR Network*) OR SU ((Inter OR multi OR interagenc*) N1 (collaborat* OR team* OR cooperation*) OR Multidisciplinar* OR Interdisciplinar* OR ((Inter OR multi) N1 (disciplinar* OR colaborat*)) OR (Collaborative N1 practice*) OR IPC OR (Chronic N1 (care OR healthcare) N1 model) OR Network*)

S4 MH "Decision Making, Shared" OR TI (SDM OR (Shared N1 decision N1 making) OR (patient N1 (centered OR centred) N1 care) OR (patient N1 (centeredness OR engagement))) OR AB (SDM OR (Shared N1 decision N1 making) OR (patient N1 (centered OR centred) N1 care) OR (patient N1 (centeredness OR engagement))) OR SU (SDM OR (Shared N1 decision N1 making) OR (patient N1 (centered OR centred) N1 care) OR (patient N1 (centeredness OR engagement)))

S5 S2 OR S3 OR S4

S6 MH "Comorbidity" OR TI (Multimorbidit* OR Comorbidit* OR (multiple N1 chronic N1 condition*) OR (Chronic N1 (illness OR disease*)) OR “Multi-morbidity” OR (multiple N1 chronic N1 condition*)) OR AB (Multimorbidit* OR Comorbidit* OR (multiple N1 chronic N1 condition*) OR (Chronic N1 (illness OR disease*)) OR “Multi-morbidity” OR (multiple N1 chronic N1 condition*)) OR SU (Multimorbidit* OR Comorbidit* OR (multiple N1 chronic N1 condition*) OR (Chronic N1 (illness OR disease*)) OR “Multi-morbidity” OR (multiple N1 chronic N1 condition*))

S7 MH "Clinical Competence+" OR MH "Aptitude" OR TI (aptitude* OR capabilit* OR competenc* OR expertise OR proficien* OR skill*) OR AB (aptitude* OR capabilit* OR competenc* OR expertise OR proficien* OR skill*) OR SU (aptitude* OR capabilit* OR competenc* OR expertise OR proficien* OR skill*)

S8 S1 AND S5 AND S6 AND S7

**Cinahl 24-01-2023**

S1 MH "Primary Health Care" OR MH "Family Practice" OR TI (((General OR Primary OR famil*) N1 (care OR healthcare OR medicine OR practice*)) OR (community N1 based)) OR AB (((General OR Primary OR famil*) N1 (care OR healthcare OR medicine OR practice*)) OR (community N1 based)) OR SU (((General OR Primary OR famil*) N1 (care OR healthcare OR medicine OR practice*)) OR (community N1 based))

S2 TI ((comprehensive OR coordinated OR Integrated OR seamless) N1 (care OR healthcare)) OR AB ((comprehensive OR coordinated OR Integrated OR seamless) N1 (care OR healthcare)) OR SU ((comprehensive OR coordinated OR Integrated OR seamless) N1 (care OR healthcare))

S3 MH "Multidisciplinary Care Team" OR TI ((Inter OR multi OR interagenc*) N1 (collaborat* OR team* OR cooperation*) OR Multidisciplinar* OR Interdisciplinar* OR ((Inter OR multi) N1 (disciplinar* OR colaborat*)) OR (Collaborative N1 practice*) OR IPC OR (Chronic N1 (care OR healthcare) N1 model) OR Network*) OR AB ((Inter OR multi OR interagenc*) N1 (collaborat* OR team* OR cooperation*) OR Multidisciplinar* OR Interdisciplinar* OR ((Inter OR multi) N1 (disciplinar* OR colaborat*)) OR (Collaborative N1 practice*) OR IPC OR (Chronic N1 (care OR healthcare) N1 model) OR Network*) OR SU ((Inter OR multi OR interagenc*) N1 (collaborat* OR team* OR cooperation*) OR Multidisciplinar* OR Interdisciplinar* OR ((Inter OR multi) N1 (disciplinar* OR colaborat*)) OR (Collaborative N1 practice*) OR IPC OR (Chronic N1 (care OR healthcare) N1 model) OR Network*)

S4 MH "Decision Making, Shared" OR TI (SDM OR (Shared N1 decision N1 making) OR (patient N1 (centered OR centred) N1 care) OR (patient N1 (centeredness OR engagement))) OR AB (SDM OR (Shared N1 decision N1 making) OR (patient N1 (centered OR centred) N1 care) OR (patient N1 (centeredness OR engagement))) OR SU (SDM OR (Shared N1 decision N1 making) OR (patient N1 (centered OR centred) N1 care) OR (patient N1 (centeredness OR engagement)))

S5 S2 OR S3 OR S4

S6 MH "Comorbidity" OR TI (Multimorbidit* OR Comorbidit* OR (multiple N1 chronic N1 condition*) OR (Chronic N1 (illness OR disease*)) OR “Multi-morbidity” OR (multiple N1 chronic N1 condition*)) OR AB (Multimorbidit* OR Comorbidit* OR (multiple N1 chronic N1 condition*) OR (Chronic N1 (illness OR disease*)) OR “Multi-morbidity” OR (multiple N1 chronic N1 condition*)) OR SU (Multimorbidit* OR Comorbidit* OR (multiple N1 chronic N1 condition*) OR (Chronic N1 (illness OR disease*)) OR “Multi-morbidity” OR (multiple N1 chronic N1 condition*))

S7 MH "Clinical Competence+" OR MH "Aptitude" OR TI (aptitude* OR capabilit* OR competenc* OR expertise OR proficien* OR skill*) OR AB (aptitude* OR capabilit* OR competenc* OR expertise OR proficien* OR skill*) OR SU (aptitude* OR capabilit* OR competenc* OR expertise OR proficien* OR skill*)

S8 S1 AND S5 AND S6 AND S7

**Trip database 24-01-2023**

| (“Integrated care” OR “Aligning policy integrated care” OR “integrated healthcare” OR “coordinated care” OR “comprehensive care” OR “seamless care”) AND (“Chronic illness” OR “chronic disease” OR “chronic condition” OR multimorbidity OR Comorbidity OR “co-occurring chronic illnesses” OR “multiple chronic condition”) AND (“Primary care” OR “primary health care” OR “General medicine” OR “General healthcare” OR “General practice” OR “Family practice” OR “family care” OR “community based”) |
| --- |
