# Supplementary material for: Primary healthcare competencies needed in the management of person-centred integrated care for chronic illness and multimorbidity: Results of a scoping review
Source: BMC Prim Care. 2023 Apr 12;24:98. doi: 10.1186/s12875-023-02050-4 (PMC10091550; doi:10.1186/s12875-023-02050-4)
Supplement: Supplementary file 3 — Supplementary Material 3 [file 12875_2023_2050_MOESM3_ESM.docx]

| Appendix: TABLE 3 CODE TREE with examples included publications | | |
| --- | --- | --- |
| Theme | **Code/Subtheme** | **Example** |
| Patient centred communication | Listening  Open communication  Clarity of communication  Motivational Interviewing  Person-centred assessment  Nonverbal communication  Equality | Listening to patients and translating their concerns to medical actions emerged as an important aspect of communication, improving the quality of care (34).  Open and respectful communication is central to  a client-centred approach (43).  Healthcare professionals should ensure communication is understood and that the patient and family’s cultural perspectives are respected (41).  Motivational Interviewing promotes provider-patient collaboration based on understanding patients’ viewpoints (33).  Patient centred interviewing improves data collection while enhancing the patient-provider relationship (30).  Primary healthcare professionals should be sensitized to identify psychosocial needs associated with medical health problems. More training is needed in communicative competencies like listening and recognizing nonverbal signals (38).  Both HCPs and patients express the importance of accessibility and communication on the basis of equality (31) |
| Interprofessional communication | Two way communication  Open communication  Equality  Effective communication | There should be a two way communication between primary healthcare professionals where each party is aware of the other’s professional backgrounds, strengths and boundaries (43).  Fostering an open communication where it is okay for everyone to have a say (23).  Reaching team consensus by dialoguing and discussing issues with all team members on an equal level and from their own perspective is highly valued (35).  Knowledge and use of effective communication techniques is a critical core competency to improve the quality of stroke decision making, as well as patient and family satisfaction and outcomes (22) |
| Collaborative teamwork | Teamwork  Sharing important information  Motivation  Shared language  Clarity of roles  Knowing each other  Trust and respect | There is a stronger cohesion between team members with different backgrounds due to complementary competence, mutual respect, open communication and equal partnership in the decision-making process, in contrast with the hierarchical structures which continue to exist in other settings (35).  Patients should be made aware of the urgency of healthcare professionals sharing relevant information with other disciplines (41).  Successful collaboration and sustaining success for the future is related to the professionals’ continuing motivation. Intrinsic motivation was described as the professionals’ personal ideological drive, need, aan willingness to collaborate (39).  Professionals from different disciplines compile an interprofessional team with an diversity of perspectives and discipline specific language. Defining a shared language could be helpful to prevent confusion in the interprofessional communication (39).  Members of interprofessional teams see the lack of understanding of different professional roles as a barrier for effective interprofessional teamwork (41).  Primary healthcare professionals believe that knowing each other well are better able to take advantage of each other’s discipline-specific competencies (39).  Trust and respect are two underlying preconditions of successful collaboration. This creates an open and safe environment in which the professionals dare to think and act broader than their own discipline (39). |
| Leadership | Team leadership  Care advocate  Team meetings | Within health systems, leadership has an important role in supporting IP teams. Key characteristics include modeling and advocating for IP teamwork, providing resources and infrastructure (environment, staff, training, incentives, etc.), and promoting shared team leadership, goals and decision-making (25).  Per patient, interprofessional team members need to consider who the appropriate case manager should be. The case manager explores goals and needs with the patient, and brings the patient’s goals and wishes as a patient advocate to the interprofessional team (39).  Comprising the planning, agenda setting, structuring, and chairing of the team meetings, is a crucial task in attaining efficient and successful team meetings (39). |
|  |  |  |
